# Underage Youth Continue to Obtain E-Cigarettes from Retail Sources in 2022: Evidence from the Truth Continuous Tracking Survey

**DOI:** 10.3390/ijerph20021399

**Published:** 2023-01-12

**Authors:** Elizabeth K. Do, Kathleen Aarvig, Emily M. Donovan, Barbara A. Schillo, Donna M. Vallone, Elizabeth C. Hair

**Affiliations:** 1 Schroeder Institute, Truth Initiative, Washington, DC 20001, USA; 2 Milken Institute School of Public Health, George Washington University, Washington, DC 20052, USA; 3 Department of Health, Behavior and Society, Johns Hopkins Bloomberg School of Public Health, Baltimore, MD 21205, USA; 4 School of Global Public Health, New York University, New York, NY 10003, USA

**Keywords:** e-cigarette, youth, young adults, source

## Abstract

(1) Background: This study aims to describe the primary sources of e-cigarettes among young people and to explore how these sources may differ by individual-level characteristics. (2) Methods: Data were obtained from a cross-sectional, continuous tracking survey of participants. The analytic sample includes current e-cigarette users (aged 15–20 years) surveyed from January to August 2022 (N = 1296). Respondents provided information on e-cigarette source of acquisition, device type, and flavors used, as well as sociodemographic and residential characteristics. Chi-square tests were used to determine differences in source of acquisition by age, gender, race/ethnicity, United States (US) census region, urban-rural classification, flavors used, and device type. (3) Results: Although most current e-cigarette users obtained their devices through a social source (56.9%), a considerable proportion obtained e-cigarettes from a retail source (43.1%). The primary retail sources of e-cigarette acquisition were vape shops (22.0%) and gas station/convenience stores (15.9%). Source of e-cigarette acquisition differed by age, gender, US census region, flavors used, and device type, such that a lower proportion of those who were younger, female, residing in the West, and used vape pens had reported obtaining e-cigarettes via retail sources. (4) Conclusions: Results indicate that a significant proportion of youth report obtaining e-cigarettes from retail sources, despite the federal, state, and local policies that prohibit the sale of any tobacco products to those under the age of 21. Comprehensive retail regulations to help restrict tobacco product access are needed to reduce e-cigarette use among young people.

## 1. Introduction

Multiple states and local municipalities prohibited the sale of electronic cigarettes (e-cigarettes) to minors under 18 years of age from 2010 through 2015. E-cigarettes are handheld, battery-powered devices that work by heating e-liquids that usually contain a mixture of nicotine, propylene glycol, vegetable glycerin, and flavorings [1]. A federal rule by the United States (US) Food and Drug Administration (FDA) extended e-cigarette sales restrictions for minors to all states in 2016 [2]. In 2019, the federal minimum legal sale age (MLSA) of tobacco products, including e-cigarettes, increased from 18 to 21 years [3]. The implementation of these policies, which were designed to reduce e-cigarette initiation and use among young people, were met with some success. From 2015 to 2019, the proportion of young people who reported buying e-cigarettes from retail stores decreased from 22.0% to 16.6% [4].

However, the start of the COVID-19 pandemic prompted most US states to issue stay-at-home orders and social distancing practices to help reduce the risk of transmission in March 2020 [5,6,7]. During this time, many non-essential retail stores, including vape shops, were ordered to close [7]. The implementation of these mandates restricted social and retail sources where young people often accessed e-cigarettes [6]. However, some young people reported that they were still able to obtain e-cigarettes via home deliveries from retailers, often without any age verification [8].

In 2021, one third of youth reported obtaining their e-cigarettes through retail sources. Specifically, 20.2% bought their e-cigarettes through vape shops or tobacco shops and 19.6% through gas stations [9]. These estimates suggest a lack of compliance with age verification processes by retailers [10,11,12,13], which allows young people to continue obtaining e-cigarette products from retail sources. Vape shops, in particular, have been found to implement less vigorous age verification methods, relative to other retail sources [11,14,15,16]. To help inform policy implementation and enforcement efforts with timely and relevant information, this study describes sources of e-cigarette acquisition among young people and illustrates how sources of e-cigarette acquisition may differ by individual-level characteristics using data from 2022.

## 2. Materials and Methods

### 2.1. Data Source and Inclusion Criteria

Data were obtained from the Truth Continuous Tracker Online (Truth CTO), a cross-sectional, continuous tracking survey of participants sourced from the national Dynata online panel (https://www.dynata.com, accessed on 11 January 2023). This survey is administered to a convenience sample of approximately 300 participants per week. Survey responses are then weighted to US census quotas according to age, race, and gender so that it is nationally representative. All study procedures were reviewed and approved for human subjects research by the Advarra Institutional Review Board. Participants or their legal guardians provided informed consent and/or assent. To be included in analyses, participants had to complete a survey between 5 January 2022 to 30 August 2022, be between 15 and 20 years old, report current e-cigarette use, and provide information on their source of e-cigarettes (n = 1296).

### 2.2. Measures

Self-reported past 30-day use of e-cigarette/vape products was used as a dichotomous measure of current use. Respondents were asked, “During the past 30 days, on how many days did you use each of the following products? JUUL; e-cigarettes/vapes (not including JUUL)”. Respondents who reported between 1 and 30 days of using either or both products were coded as current users and included in the analyses.

Source of e-cigarette acquisition was measured by the following single-response item: “Where did you get or buy the vape, e-cigarette, or JUUL device(s) that you used most recently?” Responses were categorized into the following: retail (vape shop/store that sells only e-cigarettes, internet/online, gas station/convenience store, mall or shopping center kiosk, or grocery store/drug store) and social (friend, family member, or some other person that is not a friend or family member).

Sociodemographic factors included age (15–17 years, 18–20 years), gender (male, female), race/ethnicity (non-Hispanic white, non-Hispanic Black, Hispanic/Latino, non-Hispanic Asian, and another non-Hispanic race/ethnicity), and perceived financial status (do not meet basic needs, just meet basic expenses with nothing left over, meet needs with a little left over, live comfortably).

Residential factors included US census region (East, Midwest, South, West) and urban-rural classification (urban/city area, area next to a city, small town or rural area, not sure).

Respondents were asked, “Thinking of the most recent time you used a vape or e-cigarette, what flavor(s) did you use? (Select all that apply)”. Responses included the following: tobacco; menthol; mint; clove or spice; alcoholic drink (such as wine, cognac, margarita, or other cocktails); candy, dessert, or sweets; ice; or some other flavor. Responses were recoded into the following categories: fruit, candy, dessert, or sweets only; menthol, mint, or ice only; and another flavor (e.g., tobacco, clove or spice, alcoholic drink, some other flavor, and individuals who selected more than one flavor).

To determine e-cigarette device type, respondents were asked, “What kind of e-cigarette/vape have you used in the past 30 days?” Responses included pod-based e-cigarette/vape, disposable/one-time use vapes, vape pens, tank-based vapes, and not sure. If more than one device type was selected, respondents were asked the additional question, “Which of these types of e-cigarettes/vapes did you use most recently?”

### 2.3. Statistical Analysis

Frequencies and weighted percentages are presented for sample characteristics among current e-cigarette users. Chi-square tests were used to determine whether source of acquisition differed by sociodemographic factors, residential factors, flavors used, and device type. All analyses were conducted using Stata 17 (Stata Corp, LLC; College Station, TX, USA) [17].

## 3. Results

### 3.1. Sample Characteristics

Table 1 displays the sample sociodemographic, residential, and device characteristics of current e-cigarette users. Most current e-cigarette users were aged 18–20 years (63.9%), female (53.5%), and non-Hispanic white (57.1%). Less than half (40.6%) resided in the South and reported that they lived in an area next to a city (39.4%). More than one third (34.0%) of current e-cigarette users perceived their financial status as “just meeting basic expenses with nothing left over”, while 33.4% indicated that they “meet needs with a little left over”, 20.5% indicated that they “live comfortably”, and 12.2% indicated that they “don’t meet basic expenses”.

Among current e-cigarette users, disposable e-cigarettes were the most popular e-cigarette device type (45.7%), followed by vape pens (25.4%) and pod-based e-cigarettes (19.6%). All users reported the use of flavored e-cigarette products, with 36.2% reporting use of fruit, candy, dessert, or sweet flavors only, 17.5% reporting use of menthol, mint, or ice flavors only, and 46.3% reporting use of other flavors. Although most users obtained their devices through a social source (56.9%), a considerable proportion reported obtaining their devices from retail sources (43.1%). Among those who obtained their e-cigarette via social sources, 37.7% and 13.3% reported obtaining their products through a friend or family member, respectively. Vape shops were identified as the primary retail source (22.0%), followed by gas stations/convenience stores (15.9%). Only 2.1% of current e-cigarette users purchased their e-cigarettes from the internet or online sources.

### 3.2. Differences in Demographic Characteristics, Flavors Used, and Device Type by Source of E-Cigarette Acquisition

Overall, the source of e-cigarette acquisition differed significantly across age, gender, US census region, flavors used, and device type (Table 1). A large proportion of current e-cigarette users between the ages of 15 and 17 years obtained their e-cigarette products through social sources (71.7%). More than half (51.4%) of current e-cigarette users between the ages of 18–20 years obtained their e-cigarettes from retail sources. A lower proportion of current e-cigarette users who obtained their e-cigarettes from retail sources were female (40.3% vs. 59.7%) and resided in the West (34.4% vs. 65.5%), relative to current e-cigarette users who obtained their e-cigarettes from social sources. Compared to current e-cigarette users who obtained their e-cigarettes from social sources, a greater proportion of current e-cigarette users who obtained their e-cigarettes from retail sources reported using fruit, candy, dessert, or sweet flavors (39.8% vs. 60.2%, respectively) or using menthol, mint, or ice flavors (40.1% vs. 59.9%, respectively). Nearly one third (32.7%) of vape pen users obtained their products through retail sources, while pod-based and disposable users were evenly split across social and retail sources (pod-based: 55.7% vs. 44.3%; disposable: 50.0% vs. 50.0%, respectively).

## 4. Discussion

This study presents timely information on sources of e-cigarette acquisition from January to August of 2022. Results demonstrate that although most young people reported obtaining their e-cigarettes from social sources, more than 40% of current e-cigarette users reported obtaining their e-cigarettes from retail sources. This is an increase in proportion relative to other studies conducted prior to 2022. For example, one study using data from 2019 reported that 33.6% of underage (aged 15–20 years) flavored JUUL users and 31.2% of underage users of other flavored e-cigarettes obtained their e-cigarettes from retail sources [18]. Data from the 2021 National Youth Tobacco Survey suggested that 20.2% of middle and high school students who were current users of any tobacco product obtained their products from vape shops or tobacco shops and 19.6% obtained them from gas stations or convenience stores [9]. Similar patterns are found in other countries, such as Canada, England [15], and Poland [19]. However, a lower proportion of young people in our study reported obtaining e-cigarettes via online sources, relative to other studies [18,20].

Over a quarter of e-cigarette users under the age of 18 years and nearly half of current e-cigarette users aged 18 to 20 years in our study obtained their device through a retail source. Our study and others continue to highlight that young people under the minimum legal sale age (MLSA) are purchasing e-cigarettes from retail sources, signaling potential gaps in the US Food and Drug Administration (FDA)’s enforcement of the MLSA and retailer noncompliance with federal, state, and local policies aimed at reducing e-cigarette use among young people [21,22,23].

All current e-cigarette users within our study reported use of a flavored e-cigarette product. A large proportion of e-cigarette users who obtained their devices from retail sources reported use of disposable e-cigarette devices. One explanation for this finding is the exemption in the FDA enforcement policy on unauthorized flavored cartridge-based e-cigarettes that appeal to children, including fruit and mint [24]. This FDA enforcement policy allows disposable e-cigarettes to feature flavors that are restricted in pod-based e-cigarettes [25]. Another possible explanation for this finding could be the difference in cost, as disposables are generally less expensive than other device types (e.g., pod-based and tanks) that require purchasing costly startup kits [26].

Comprehensive efforts are needed to strengthen retailer compliance with federal, state, and local policies. Establishing assurances of voluntary compliance (AVCs) is one way to strengthen policy compliance. AVCs are legally binding standards and practices designed to reduce the appeal of tobacco products and advertisements that target youth [19]. Common examples of AVCs include (1) refraining from using advertising that targets and appeals to youth; (2) prohibiting the sale of look-alike tobacco products; (3) installing and using cash registers that require store clerks to enter in the birth date of customers before completing tobacco sales; (4) agreeing to use an independent agency to perform unannounced compliance checks on retailer stores; (5) agreeing not to hire anyone under age 18 to sell tobacco products; and (6) training employees on relevant federal, state, and local laws regarding the MLSA and consequences of noncompliance.

Our timely results also point to the need for comprehensive surveillance to improve the efficacy of youth access policies. For example, further evaluation efforts across jurisdictions with variation in e-cigarette policy are needed to (1) assess product availability, marketing, and pricing across physical and online retailers; (2) determine the level of retailer compliance with existing federal, state, and local policies; (3) monitor how the tobacco industry responds to the implementation of federal, state, and local regulations and how this influences the actions of e-cigarette retailers and young people; and (4) identify ways in which consumers under the minimum legal sale age respond to changes in e-cigarette related policy [27].

### Limitations

Study limitations include the study design, which includes pooled convenience samples of repeated cross-sectional surveys. This study design does not allow for causal inference and relies on self-reported data that may be influenced by social desirability and/or recall bias. Despite these limitations, the current findings highlight the need for timely information to help improve the efficacy of existing and future federal, state, and local policies.

## 5. Conclusions

Young people—including underage youth—continue to obtain e-cigarettes through retail stores, including vape shops, gas stations, and convenience stores in 2022. Federal, state, and local policies need to establish and enforce comprehensive retail regulations to help restrict tobacco product access and, in turn, reduce e-cigarette use among young people.

## Figures and Tables

**Table 1:** Sociodemographic, residential, and device type characteristics by source of acquisition among current e-cigarette users (5 January 2022–30 August 2022).

	Total (n = 1296)		Retail (n = 559, 43.1%)		Social (n = 737, 56.9%)		Chi-Square, *p*-Value
	N	%	N	%	N	%	
**Age (years)**							*64.1, <0.001*
15–17	463	36.1	131	28.3	332	71.7	
18–20	833	63.9	428	51.4	405	48.6	
**Gender**							*4.7, 0.029*
Male	594	46.5	276	46.3	318	53.7	
Female	702	53.5	283	40.3	419	59.7	
**Race/Ethnicity**							1.8, 0.1250
White, Non-Hispanic	712	57.1	321	45.0	391	55.0	
Black, Non-Hispanic	132	10.6	64	47.9	68	52.1	
Hispanic	280	22.0	105	37.6	175	62.4	
Asian, Non-Hispanic	58	4.7	23	39.6	35	60.4	
Another race/ethnicity	71	5.7	26	36.6	45	63.4	
**United States Census Region**							*3.6, 0.014*
East	259	20.0	123	47.4	136	52.6	
Midwest	303	23.5	123	40.6	180	59.4	
South	528	40.6	242	45.8	286	54.2	
West	206	15.8	71	34.4	135	65.6	
**Urban-Rural Classification**							2.6, 0.050
Urban/city area	364	28.1	161	44.1	203	55.9	
Area next to a city	511	39.4	239	46.7	272	53.3	
Small town or rural area	382	29.5	143	37.5	239	62.5	
Not sure	39	3.0	16	41.1	23	58.9	
**Perceived Financial Status**							0.4, 0.775
Don’t meet basic expenses	158	12.2	64	40.4	94	59.6	
Just meet basic expenses with nothing left over	441	34.0	197	44.7	244	55.3	
Meet needs with a little left over	433	33.4	188	43.2	245	56.8	
Live comfortably	264	20.5	110	41.8	154	58.2	
**Most Recent E-cigarette device type**							*7.9, <0.001*
Pod-based	254	19.6	113	44.3	141	55.7	
Disposable	591	45.7	296	50.0	295	50.0	
Vape pen	330	25.4	108	32.7	222	67.3	
Tank	64	5.0	26	40.6	38	59.4	
Not sure	57	4.4	16	28.0	41	72.0	
**E-cigarette flavor**							*6.3, 0.044*
Fruit/candy/dessert only	469	36.2	282	60.2	187	39.8	
Menthol/mint/ice only	227	17.5	136	59.9	91	40.1	
Another flavor (tobacco, alcoholic, clove/ spice, some other flavor, or indicated use of more than one flavor)	600	46.3	319	53.2	281	46.8	
**Source of E-cigarette Acquisition**							N/A
Friend	487	37.7	N/A	N/A	487	37.7	
Family member	173	13.3	N/A	N/A	173	13.3	
Another person (not a friend or family member)	77	5.9	N/A	N/A	77	5.9	
Vape shop	286	22.0	286	22.0	N/A	N/A	
Gas station/convenience store	206	15.9	206	15.9	N/A	N/A	
Grocery store	21	1.6	21	1.6	N/A	N/A	
Internet/Online	27	2.1	27	2.1	N/A	N/A	
Mall, shopping center kiosk, or some other place	19	1.5	19	1.5	N/A	N/A	

Note. Reported % under “Retail” and “Social” columns are row percentages for age, gender, race/ethnicity, and most recent e-cigarette device type. Reported % under “Total” column and across e-cigarette source are column percentages. N/A = not applicable. *Italics* indicate statistical significance at *p*-value < 0.05.

## Data Availability

Data sharing is not applicable to this article.
